# Lung transplant outcomes in myositis, systemic sclerosis and idiopathic pulmonary fibrosis: a multicentre retrospective analysis

**DOI:** 10.1093/rheumatology/keag200

**Published:** 2026-04-15

**Authors:** Angela Chang, Navid Saleh, Alec Yu, Darya S Jalaledin, Sabrina Anh-Tu Hoa, Robert D Levy, Jennifer M Wilson, Charles D Poirier, James Choi, John Yee, Océane Landon Cardinal, Hyein Kim, Kun Huang

**Affiliations:** Department of Medicine, University of British Columbia, Vancouver, BC, Canada; Department of Medicine, University of British Columbia, Vancouver, BC, Canada; Division of Rheumatology, University of British Columbia, Vancouver, BC, Canada; Division of Rheumatology, Centre hospitalier de l’Université de Montréal, Montreal, Quebec, Canada; Division of Rheumatology, Centre hospitalier de l’Université de Montréal, Montreal, Quebec, Canada; Arthritis Research Canada, Vancouver, BC, Canada; Division of Respirology, Vancouver General Hospital, Vancouver, BC, Canada; Division of Respirology, Vancouver General Hospital, Vancouver, BC, Canada; Lung Transplant Program, Centre hospitalier de l’Université de Montréal, Montreal, Quebec, Canada; Division of Thoracic Surgery, Vancouver General Hospital, Vancouver, BC, Canada; Division of Thoracic Surgery, Vancouver General Hospital, Vancouver, BC, Canada; Division of Rheumatology, Centre hospitalier de l’Université de Montréal, Montreal, Quebec, Canada; Division of Rheumatology, University of British Columbia, Vancouver, BC, Canada; Arthritis Research Canada, Vancouver, BC, Canada; Division of Rheumatology, University of British Columbia, Vancouver, BC, Canada; Arthritis Research Canada, Vancouver, BC, Canada; Division of Rheumatology, Department of Medicine, Surrey Memorial Hospital, Surrey, BC, Canada

**Keywords:** inflammatory myopathy, systemic sclerosis, interstitial lung disease, idiopathic pulmonary fibrosis, lung transplant

## Abstract

**Objectives:**

To describe clinical characteristics and post-lung transplant outcomes of patients with idiopathic inflammatory myopathies (IIM), SSc and idiopathic pulmonary fibrosis (IPF).

**Methods:**

We retrospectively analysed interstitial lung disease (ILD) patients with IIM (*n* = 22), SSc (*n* = 32) and IPF (*n* = 64) who underwent lung transplantation (2012–24) at two Canadian centres, Vancouver and Montréal.

**Results:**

Among IIM patients, 41% were clinically amyopathic at presentation, and 45% had anti-melanoma differentiation-associated protein 5 (anti-MDA5) DM, all with rapid progressive (RP)-ILD, 32% anti-synthetase syndrome, 14% overlap myositis and 9% other DM. In SSc, 88% had pulmonary hypertension (PH) (31% severe) and 78% had oesophageal dysmotility. IIM patients required more frequent pre-transplant intensive care unit (ICU) admission and emergency transplantation. Post-transplant, IIM patients had longer ICU/hospital stays. There were no significant differences in 1-year survival, survival at last follow-up (median: 2.8 years for IIM, 2.5 years for SSc and 3.6 years for IPF), incidence of chronic lung allograft dysfunction or malignancy. Subgroup analyses of IIM [stratified by transplant urgency, extracorporeal membrane oxygenation (ECMO) support and amyopathy] and SSc (stratified by severe PH, oesophageal dysmotility and transplant urgency) showed no significant differences in long-term survival. No autoimmune disease recurrence was observed.

**Conclusion:**

Despite their underlying autoimmune diseases, post-transplant survival outcomes of selected IIM and SSc patients did not differ significantly from those with IPF. IIM patients with RP-ILD necessitating emergency transplantation and ECMO support exhibited survival similar to those without such complications. However, their more complex pre- and post-transplant courses emphasize the necessity for individualized lung transplant strategies and a multidisciplinary management approach.

Rheumatology key messages

- Idiopathic inflammatory myopathies (IIM) patients have more peri-transplant complications than patients with SSc and idiopathic pulmonary fibrosis (IPF).
- Post-lung transplant, survival in IIM patients was not significantly different from SSc and IPF.
- Survival was similar for anti-MDA5 patients, even those requiring emergency transplant and circulatory support.

## Introduction

Interstitial lung disease (ILD) is a common manifestation of systemic autoimmune rheumatic diseases (SARDs), occurring in 44–50% of patients with SSc and 33–50% with idiopathic inflammatory myopathies (IIM) [1]. Despite advancements in antifibrotic and immunosuppressive therapies, ILD remains a significant cause of morbidity and mortality in both IIM and SSc, often leading to progressive respiratory failure [2, 3].

Lung transplantation may be an option when lung function declines despite optimal medical therapy and is a well-established intervention for advanced idiopathic pulmonary fibrosis (IPF) [4]. However, patients with IIM and SSc have complex extrapulmonary manifestations of their disease, which limit the extrapolation of lung transplant outcomes data from IPF cohorts. Specifically, diaphragmatic muscle weakness and dysphagia in IIM, and oesophageal dysfunction, small intestinal bacterial overgrowth and pulmonary hypertension (PH) in SSc may increase perioperative risks and postoperative complications [5–7]. Moreover, chronic CS and immunosuppressive therapies in IIM and SSc, combined with an often-poor nutritional status, may impact lung transplant outcomes [8]. Despite these concerns, post-transplant survival is reported to be similar between SARD-ILD and IPF in observational studies [8–12]. To date, there has been no study comparing lung transplant outcomes of patients with IIM versus another SARD, such as SSc, or a non-SARD control, such as IPF.

To address this knowledge gap, we conducted a multicentre retrospective cohort study to describe the baseline characteristics of patients with IIM who underwent lung transplantation and compare post-transplant survival and complications versus those with SSc and IPF.

## Patients and methods

### Study design

We conducted a retrospective review of all double lung transplant recipients with ILD as the indication for transplantation between 1 January 2014 and 30 April 2024, at the Vancouver General Hospital (British Columbia, Canada), and between 1 January 2012 and 31 December 2024, at the Centre hospitalier de l’Université de Montréal (Quebec, Canada) ILD clinic. These institutions are the sole lung transplant centres in their respective provinces in Canada. In Canada’s universal healthcare system, all costs and resources associated with pre- and post-transplant care are publicly funded.

### Cohort definition

Patients with IIM or SSc, as defined by the 2017 ACR/EULAR [6] and 2013 ACR/EULAR [13] classification criteria, respectively, were reviewed and confirmed by rheumatologists (K.H. and H.K. in BC; O.L.C. and S.A.-T.H. in QC). We excluded patients with pulmonary arterial hypertension as the primary indication for transplantation (e.g. those with SSc). The IPF cohort comprised all patients diagnosed with IPF by multidisciplinary review before transplantation. Suitability for lung transplantation was determined by the multidisciplinary transplant team after a comprehensive guideline-based assessment [14, 15]. Standard perioperative and post-transplant care was delivered according to international guidelines [16, 17]. All patients with SSc from BC transplant centre (*n* = 23) were managed with post-pyloric enteral nutrition for 3–6 months, irrespective of their oesophageal dysmotility status.

### Data collection

Clinical, laboratory, radiographic and pathological data were retrospectively reviewed and verified by a multidisciplinary team comprising ILD respirologists, rheumatologists, radiologists and a thoracic surgeon specializing in lung transplantation. The primary outcome was long-term survival, defined as the time from lung transplantation to death or last follow-up.

Baseline variables included age at ILD diagnosis, sex, smoking status, Charlson Comorbidity Index, pre-transplant BMI, autoantibodies, extrapulmonary manifestations, radiological findings, medication history, pre-transplant forced vital capacity (FVC) (Global Lung Function Initiative 2012 equations) [18], diffusion capacity for carbon monoxide (DLCO) and pulmonary artery pressures [pulmonary artery systolic pressure/mean pulmonary arterial pressure (PASP/mPAP)]. The term ‘clinically amyopathic DM’ describes patients with normal muscle strength by Medical Research Council scale, regardless of creatine kinase levels. PH is defined as mPAP >20 mmHg [19]. Severe PH is defined as mPAP ≥35 mmHg or mPAP ≥25 mmHg with a cardiac index ≤2.0 l/min/m^2^ [20], acknowledging that this definition has evolved over time [21, 22]. While most patients with IIM, SSc and IPF underwent right heart catheterization (RHC) as per pre-lung transplant guidelines, the haemodynamic data were not consistently available on chart review. Our PH category in SSc comprises a mixture of pure group 3 and cases of combined group 3/1 PH. Rapid progressive (RP)-ILD was defined as displaying two or more of the following within 3 months: (i) dyspnoea exacerbation; (ii) an increase in parenchymal abnormality on chest CT; and (iii) one of the following physiological changes: >10% decrease in vital capacity or > 1.33 kPa decrease in arterial oxygen tension [23]. An emergency transplant is defined as an unplanned hospital admission resulting in transplantation, and the need for intensive care unit (ICU) admission, mechanical ventilation or extracorporeal membrane oxygenation (ECMO).

Post-transplant variables included age, ILD duration, CMV mismatch, ICU/hospital stay, 1-year data on survival, FVC/DLCO, rejection episodes, infections and CMV viraemia, plus final follow-up on survival, chronic lung allograft dysfunction (CLAD) and malignancies (excluding non-melanoma skin cancers). Among all participants, only one SSc patient required redo transplantation at 1 year (for acute rejection/CLAD). For this case, the 1-year follow-up data corresponded to the first transplant, while the final follow-up data reflected outcomes following the second transplant.

### Statistical analysis

Continuous variables were analysed using the Kruskal–Wallis test. Categorical variables were compared using the Chi-squared test. Kaplan–Meier survival curves were generated for the IIM, SSc and IPF groups, and differences in survival distributions were assessed using the log-rank test. Poisson regression was used to compare the incidence rates of CLAD and post-transplant malignancy among the three groups. Statistical analyses were conducted using R software (version 4.4.3; R Core Team, Vienna, Austria). A two-tailed *P*-value of <0.05 was considered statistically significant.

### Ethical considerations

This study conforms with the principles of the Declaration of Helsinki with institutional Research Ethics Boards (VGH, #H24-01298 and CHUM, #2021-9043) approval. The need for individual consent was waived in accordance with institutional guidelines.

## Results

### Baseline patient characteristics

A total of 22 patients with IIM, 32 with SSc and 64 with IPF were included. The clinical characteristics and serologic profiles of IIM and SSc patients at diagnosis are summarized in Supplementary Fig. S1 and Table S1. Among the 22 IIM patients, the most common extrapulmonary features at the time of diagnosis were elevated creatine kinase levels (73%), inflammatory polyarthritis (73%) and DM-associated rash (73%). Ten (45%) were anti-melanoma differentiation-associated protein 5 (anti-MDA5) DM, all of whom presented with RP-ILD. Seven patients (32%) were diagnosed with anti-synthetase syndrome (ASyS), three (14%) overlap myositis and two (9%) unspecified DM. Nine patients (41%) demonstrated clinically amyopathic phenotypes, including four with anti-MDA5 DM. Among the 32 patients with SSc, RP was universal (100%), followed by PH (88%), sclerodactyly (84%) and gastroesophageal reflux disease (84%). Serologic testing revealed anti-Scl-70 antibodies in 12 (38%), anti-CENP-B in 2 (6%) and anti-RNA Pol III in 1 (3%). Cutaneous involvement included limited disease in 23 (72%), diffuse disease in 8 (25%) and SSc sine scleroderma in 1 (3%). While RHC was performed in most patients with SSc and IPF and in a subset of those with IIM, many of the corresponding haemodynamic data were missing. RHC haemodynamics were available in 28 of the 32 SSc patients. The remaining four patients underwent echocardiography, which showed no suspicion of PH. PH was diagnosed in 28 SSc patients (88%), with 10 severe [median mPAP of 44 mmHg, interquartile range (IQR) 39–47] and 18 non-severe (median mPAP 24 mmHg, IQR 21–26). Within the severe PH group, eight had mixed group 3/1 PH, and two had group 3 PH only.

Baseline pre-transplant clinical characteristics of the IIM, SSc and IPF cohorts are summarized in Table 1. Patients with IIM and SSc were significantly younger than those with IPF at both the time of ILD diagnosis and at the time of lung transplantation. Additionally, IIM and SSc patients were more likely to be females and had fewer comorbidities compared with patients with IPF. By the time of lung transplant evaluation, no patient with IIM including those with anti-MDA5 DM demonstrated active myopathy or ulcerative rash.

**Table 1:** Baseline patient characteristics pre-transplant.

Variables	IIM (*n* = 22)	SSc (*n* = 32)	IPF (*n* = 64)	*P*-value
Demographics				
Age at SARD diagnosis, median (IQR)	47 (37–52)	46 (37–56)	NA	0.77
Age at ILD diagnosis, median (IQR)	47 (41–52)	49 (40–56)	60 (58–64)	**<0.001**
Age at lung transplant, median (IQR)	54 (48–59)	57 (44–60)	65 (62–68)	**<0.001**
Female, n (%)	13 (59)	22 (69)	11 (17)	**<0.001**
White, n (%)	15 (68)	21 (66)	51 (80)	0.27
Ever smoker, n (%)	12 (55)	14 (44)	47 (73)	**0.014**
BMI, median (IQR)	28 (22–32)	25 (22–30)	28 (24–29)	0.49
Charlson Comorbidity Index, median (IQR)	2 (2–3)	2 (1–3)	3 (2–4)	**<0.001**

*P*-values in bold text indicate statistical significance. IIM: idiopathic inflammatory myopathies; ILD: interstitial lung disease; IPF: idiopathic pulmonary fibrosis; IQR: interquartile range; SARD: systemic autoimmune rheumatic disease; NA: not applicable.

### Pre-transplant variables

Table 2 summarizes key clinical variables prior to lung transplantation. Compared with patients with IPF, those with SSc had significantly lower DLCO, higher PASP and a longer duration of ILD.

**Table 2:** Clinical variables pre-transplant.

Variables at transplant	IIM (*n* = 22)	SSc (*n* = 32)	IPF (*n* = 64)	*P*-value
FVC (% of predicted), median (IQR)	50 (40–59)	48 (43–58)	51 (39–64)	0.79
DLCO (% of predicted), median (IQR)	36 (24–43)	23 (19–32)	31 (24–38)	0.074
PASP (mmHg), median (IQR)	40 (33–46)	43 (37–64)	35 (31–42)	**0.012**
SARD diagnosis to transplant (years), median (IQR)	3.9 (0.3–12.2)	6.1 (3.5–11.1)	NA	0.28
ILD diagnosis to transplant (years), median (IQR)	3.2 (0.2–10.1)	5.8 (3.7–7.3)	3.9 (2.3–5.8)	**0.021**
Prior use of immunosuppressants, n (%)	22 (100)	32 (100)	4 (6)	**<0.001**
Prior use of antifibrotic, n (%)	1 (5)	1 (3)	41 (64)	**<0.001**
ICU admission prior to transplant, n (%)	10 (45)	4 (13)	9 (14)	**0.007**
Mechanical ventilation, n (%)	10 (45)	3 (9)	4 (6)	**<0.001**
ECMO, n (%) (types)	9 (41) (8 VV, 1 VA)	2 (6) (2VV)	3 (5) (3VV)	**<0.001**
Emergency transplant, n (%)	11 (50)	4 (13)	15 (23)	**0.025**

*P*-values in bold text indicate statistical significance. DLCO: diffusing capacity of the lung for carbon monoxide; ECMO: extracorporeal membrane oxygenation; FVC: forced vital capacity; ICU: intensive care unit; IIM: idiopathic inflammatory myopathies; ILD: interstitial lung disease; IPF: idiopathic pulmonary fibrosis; IQR: interquartile range; PASP: pulmonary artery systolic pressure; SARD: systemic autoimmune rheumatic disease.

Use of immunosuppressive therapy was more prevalent in the IIM and SSc cohorts, whereas antifibrotic agents were more commonly prescribed in the IPF group. In the IIM cohort, the median number of immunosuppressive agents used prior to transplant was 2.5 (IQR 1–4). The most frequently prescribed immunosuppressant was MMF (82%, *n* = 18), followed by CYC and rituximab (41% each, *n* = 9), and tacrolimus and AZA (23% each, *n* = 5). IVIG was used in 31% (*n* = 7) of IIM patients. In the SSc cohort, the median number of pre-transplant immunosuppressive agents was 1.5 (IQR 1–2). MMF was the most common (91%, *n* = 29), followed by AZA (22%, *n* = 7), and CYC and rituximab (19% each, *n* = 6).

Patients with IIM were more likely to require ICU admission, mechanical ventilation, ECMO support and emergency transplantation compared with those with IPF (Table 2). Among the IIM cohort, 11 patients (50%) underwent emergency transplantation due to RP-ILD, including 10 with anti-MDA5 DM and 1 with ASyS. IIM patients also experienced longer durations of mechanical ventilation and had higher rates of renal replacement therapy and sepsis (Supplementary Table S2). Of the 10 IIM patients (45%) admitted to the ICU prior to transplantation, all required mechanical ventilation, with a median duration of 23 days, and 9 (41%) required ECMO, with a median duration of 19 days. In the SSc cohort, four patients (13%) required ICU admission, three (9%) mechanical ventilation and two (6%) ECMO. Among IPF patients, nine (19%) were admitted to the ICU, with four (6%) requiring mechanical ventilation and three (5%) ECMO.

### Post-transplant outcomes

The post-transplant anti-rejection medications are outlined in Supplementary Table S3. Tables 3 and 4 summarize the short- and long-term post-transplant outcomes. In the immediate post-transplant period, patients with IIM and SSc required prolonged ICU stays compared with those with IPF (median durations: 33 days for IIM, 9 days for SSc and 4 days for IPF). Similarly, the median hospitalization duration was longer for IIM patients (59 days for IIM, 31 days for SSc and 22 days for IPF).

**Table 3:** One year post-transplant outcomes.

1-year outcomes	IIM (*n* = 22)	SSc (*n* = 32)	IPF (*n* = 64)	*P*-value
Post-transplant ICU days, median (IQR)	33 (12–50)	9 (3– 23)	4 (3–9)	**<0.001**
Post-transplant hospitalization days, median (IQR)	59 (31–112)	31 (20–42)	22 (17–31)	**<0.001**
Alive at 1 year, n (%)	20 (91)	27 (84)	59 (92)	0.48
FVC (% of predicted), median (IQR)	64 (50–86)	63 (57–75)	84 (67–99)	**0.001**
DLCO (%of predicted), median (IQR)	56 (41–62)	57 (47.5–63.5)	61 (45–75)	0.28
Acute rejection, n (%)	6 (27)	12 (38)^a^	31 (48)	0.51
Pneumonia requiring hospitalization, n (%)	8 (36)	14 (44)	20 (31)	0.48
Non-pulmonary infection requiring hospitalization, n (%)	7 (32)	13 (41)	14 (22)	0.084
Fungal infection requiring therapy, n (%)	4 (18)	11 (34)	22 (34)	0.28
CMV viremia requiring therapy, n (%)	6 (27)	7 (22)	15 (23)	0.90

*P*-values in bold text indicate statistical significance. ^a^One of the acute rejections resulted in redo double lung transplant. DLCO: diffusing capacity of the lung for carbon monoxide; FVC: forced vital capacity; ICU: intensive care unit; IIM: idiopathic inflammatory myopathies; IPF: idiopathic pulmonary fibrosis; IQR: interquartile range.

**Table 4:** Post-transplant outcomes at the last follow-up.

Long-term outcomes	IIM (*n* = 22)	SSc (*n* = 32)	IPF (*n* = 64)	*P*-value
Follow-up duration (years), median (IQR)	2.8 (1.8–5.5)	2.5 (1.2–5)	3.6 (2.1–6.2)	0.18
Alive at last follow-up, n (%)	15 (68)	25 (78)	42 (66)	0.87
CLAD, rate per 100 person years	2.46	1.92	6.58	
CLAD, rate ratio vs IPF (95% CI)	0.37 (0.09–1.49)	0.29 (0.07–1.17)	Ref	IIM-IPF: 0.21; SSc-IPF: 0.08
Post-transplant malignancy (excluding non-melanoma skin cancer), rate per 100 person years	1.23	0.96	4.38	
Post-transplant malignancy, rate ratio vs IPF (95% CI)	0.28 (0.04–2.11)	0.22 (0.03–1.70)	Ref	IIM-IPF: 0.22; SSc-IPF: 0.14

CLAD: chronic lung allograft dysfunction; IIM: idiopathic inflammatory myopathies; IPF: idiopathic pulmonary fibrosis; IQR: interquartile range.

At 1 year post-transplant, survival rates were 91% for IIM, 84% for SSc and 92% for IPF patients, with no significant differences between groups. Both IIM and SSc patients had significantly lower FVC compared with IPF patients (median FVC: 64% for IIM, 63% for SSc and 84% for IPF), although DLCO did not differ across the groups. No significant differences were observed in the rates of acute rejection, pneumonia requiring hospitalization, non-pulmonary infections, fungal or systemic CMV infections. Two deaths occurred within 1 year post-transplant in IIM: one due to ischemic colitis and the other to multiorgan failure. Five deaths occurred within the first year in SSc, most commonly organ failure, including heart failure (*n* = 2), COVID-19 pneumonia (*n* = 1) and sepsis-induced multisystem failure (*n* = 1). One patient died of intracranial haemorrhage secondary to unrecognized infectious endocarditis with mycotic brain aneurysms (*n* = 1).

**Figure 1:**
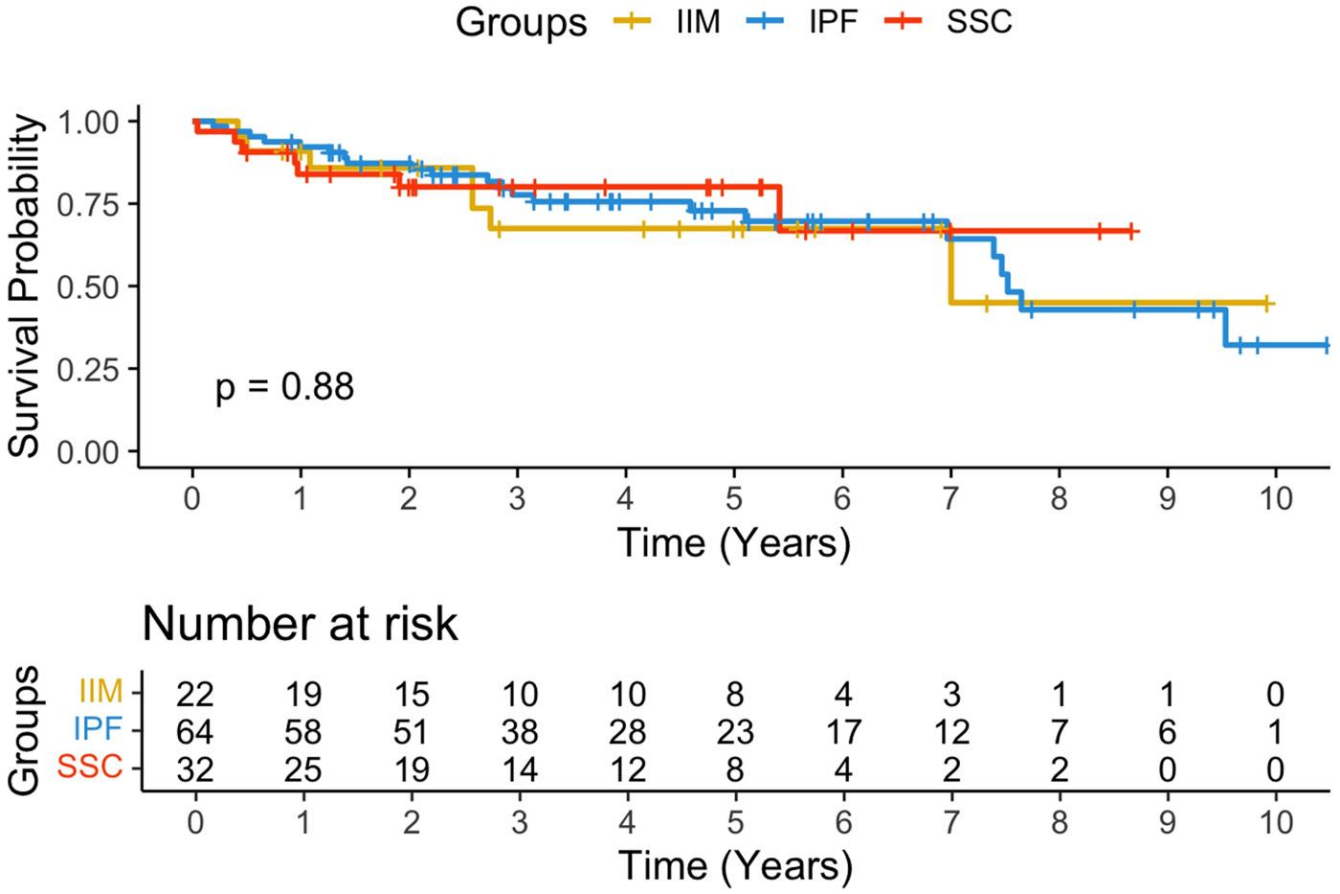
Kaplan–Meier overall survival curve of IIM, SSc and IPF patients

At the last available post-transplant follow-up (median: 2.8 years for IIM, 2.5 years for SSc and 3.6 years for IPF), Kaplan–Meier analysis showed no differences in survival (Fig. 1). Among IIM patients, subgroup analyses revealed no statistically significant differences in long-term survival for emergency *vs* non-emergency transplant (64% *vs* 73%), ECMO supported *vs* non-ECMO supported recipients (67% *vs* 69%), and amyopathic *vs* myopathic IIM subtypes (69% *vs* 67%) (Supplementary Fig. S2). Similarly, among SSc patients, long-term survival did not differ statistically when stratified by severe PH (70% *vs* 82%), oesophageal dysmotility (76% *vs* 86%) and emergency *vs* non-emergency transplant (100% *vs* 75%) (Supplementary Fig. S3).

CLAD developed in 2 (9.1%) IIM, 2 (6%) SSc and 18 (28%) IPF patients. No statistically significant differences were observed in the rates of CLAD or malignancy among the three groups (Table 4). Notably, 13 of 32 SSc patients received a single intravenous dose of methylprednisolone (500–1000 mg) as part of induction immunosuppression and desensitization protocols. None of these patients developed scleroderma renal crisis following transplant.

At final follow-up, five additional deaths occurred in IIM all due to infections (three pneumonia, one bowel perforation and one undefined source). In the SSc cohort, two further deaths resulted from respiratory failure (one acute rejection after re-transplant, one COVID-19). Only one SSc patient underwent redo transplant (for acute rejection/bronchiolitis obliterans syndrome–CLAD) but later developed recurrent acute rejection and respiratory failure, precluding further intervention due to frailty and graft dysfunction.

In both the IIM and SSc cohorts, no patients demonstrated clinical, biochemical or radiographic evidence suggestive of post-transplant autoimmune disease recurrence in any organ system.

## Discussion

The complex and severe extrapulmonary manifestations of IIM and SSc, including proximal/respiratory/oropharyngeal muscle weakness, severe gastrointestinal (GI) dysmotility, malnutrition and scleroderma renal crisis, raise significant concerns about post-transplant survival. These factors, compounded by chronic CS exposure and immunosuppression requirements, have made many centres hesitant to pursue lung transplant in these populations. This multicentre study presents the first descriptive analysis of post-transplant outcomes among IIM patients *vs* two groups: SSc, representing another SARD, and IPF, serving as a non-SARD comparator. While survival, risk of acute and chronic rejection, infections, and post-transplant malignancies were comparable across the three groups, distinctive differences were observed.

IIM and SSc patients had lower comorbidity burdens than IPF patients, attributable to their younger age at ILD diagnosis (by 11–13 years) and transplantation (by 8–11 years), and fewer smokers. Compared with the largest previous European IIM-ILD transplant study (*n* = 64) [8], our cohort had similar age and survival but featured more female patients (72% *vs* 45%), anti-MDA5 DM (45% *vs* 20%), RP-ILD (50% *vs* 39%) and emergency transplants (50% *vs* 33%). We observed lower rates of CLAD (9% *vs* 23%) and post-transplant IIM relapse (0% *vs* 8%). Unlike the European study, we found no survival difference between myopathic and amyopathic IIM patients (69% *vs* 67%). In contrast to Khan *et al.*’s study reporting 67% 3-year mortality in IIM-ILD (only two of six surviving) [24], our IIM cohort demonstrated 32% mortality at 2.8 years. This discrepancy remains unexplained, particularly since none of Khan’s IIM patients required pre-transplant mechanical ventilation. Khan reported higher CLAD rates in IIM *vs* other SARDs, whereas we found comparable low CLAD rates between IIM and SSc. These contrasting findings highlight the need for larger, prospective multicentre studies to elucidate the factors influencing post-transplant outcomes in IIM-ILD.

Our group previously reported favourable post-transplant outcomes in anti-MDA5 DM–associated RP-ILD [25, 26]. The findings in this study support the evolving approach of offering transplants to anti-MDA5 DM patients, historically considered poor candidates. RP-ILD affected 38% of our Canadian anti-MDA5 cohort [27], often necessitating ICU care and ECMO. Despite longer post-transplant ICU/hospital stays in IIM patients, likely due to higher emergency transplant (50%) and pre-transplant ICU admission (45%) rates, long-term survival was comparable to SSc and IPF cohorts. Furthermore, survival was similar between emergency and non-emergency IIM transplants, and between ECMO- and non-ECMO-supported cases, though these findings require cautious interpretation given small sample sizes. Notably, the survival outcome in our ECMO-supported IIM patients (67%) is consistent with published data, including a multicentre case series of 22 IIM-ILD patients on ECMO, which reported a survival rate of 64% [28].

ASyS-associated ILD typically progresses more slowly than the rapidly evolving ILD seen in anti-MDA5 DM [29]. Only 1 of 7 ASyS patients (14%) required emergency transplant, whereas all 10 anti-MDA5 patients presented with RP-ILD requiring emergency transplant, These findings are consistent with the study by Rivière *et al*. which reported emergency transplant in 85% of anti-MDA5 DM patients compared with only 18% of those with ASyS [8].

Our SSc cohort differed from published studies [12, 30, 31], with older recipients (median 57 years), more limited skin sclerosis (72%) and comparable 1-year survival (84%) but numerically superior long-term survival (78% *vs* 68%). Despite 88% having PH (severe in 31%), survival was comparable to IPF patients, and PH severity did not impact survival outcomes, possibly due to exclusive bilateral transplantation which may result in greater reductions in pulmonary arterial pressure and improved functional outcomes [16, 32]. Oesophageal dysmotility did not affect survival in our SSc patients, possibly due to the BC practice of post-pyloric tube feeding for 3–6 months post-transplant to limit aspiration risk, though this approach is not standardized [16, 17]. While not statistically significant, more SSc patients (41%) experienced non-pulmonary infections (predominantly GI), all with documented dysmotility, including incompetent lower oesophageal sphincter, dilatation or suspected bacterial overgrowth. Overall, comparable outcomes between SSc and IPF cohorts suggest that SSc-ILD, even with severe PH, should not be a contraindication to lung transplant in appropriately selected candidates.

Recurrence of SARDs in the transplanted organ is often a concern during transplant evaluation. In our cohort, we observed no recurrence of IIM- or SSc-associated ILD following lung transplantation, with a median follow-up of 2.8 and 2.5 years, respectively. Previous studies have reported clinical relapse, primarily mild myositis and cutaneous manifestations, in ∼8% of lung transplant recipients with IIM; however, these relapses were mild, and no cases of ILD recurrence in the allograft were observed [8]. Similarly, multiple cohort studies have found no evidence of SSc recurrence in the lung allograft following transplant [12, 33, 34], likely due to the protective effect of maintenance immunosuppressive therapy which also controls underlying SARD activity.

Inter-centre variability exists in the listing practices for SARD patients undergoing lung transplant assessment. In response, the International Society for Heart and Lung Transplantation (ISHLT) established standardized guidelines through its 2021 consensus document, which addresses two critical components: (i) disease-specific extrapulmonary manifestations requiring comprehensive evaluation and (ii) absolute contraindications to transplantation [14]. In contrast to the guideline, at the BC Transplant Center, severe achalasia or complete aperistalsis is not considered a contraindication for lung transplant in SSc patients. For BC SSc patients, it is standard practice to provide post-pyloric enteral nutrition for 3–6 months post-transplant, an approach commonly used across transplant centres [17]. For IIM, essential assessments focus on malignancy screening and evaluation for cardiac, musculoskeletal and GI involvement. Formal guidelines in contraindication remain undefined, with active/refractory myositis (particularly with cardiac involvement) generally precluding lung transplant candidacy [14].

This study has several important limitations to consider. First, the retrospective design introduces the potential for selection bias and missing data, even with our rigorous verification efforts. Documentation of the severity of important extrapulmonary manifestations (e.g. diaphragmatic weakness, oesophageal dysmotility and aspiration) was often incomplete. Second, the study’s generalizability may be limited by regional demographic differences observed between cohorts. Notably, in our study, the BC IIM group had a substantial proportion of Asian patients (31%), while this demographic was absent in the Quebec cohort, potentially reflecting distinct population characteristics. Additionally, the predominance of anti-MDA5 DM (45%) and RP-ILD cases in our IIM cohort may also limit applicability to the broader spectrum of myositis-associated ILD, particularly more chronic progressive phenotypes. Finally, only 15 (47%) SSc patients had documented SSc-specific autoantibodies, and only 5 (21%) underwent extended scleroderma antibody panel testing, including anti-RNA polymerase III antibodies. As a result, the full extent of SSc-specific autoantibody profiles may not have been captured in this cohort.

Despite the above limitations, this multicentre study provides the first direct comparative evidence that SARD-ILD subtypes, particularly high-risk groups like anti-MDA5 DM with RP-ILD, can undergo successful lung transplant. The similar long-term survival across groups, despite significantly longer ICU stays and hospitalizations in IIM patients, supports a paradigm shift toward considering these populations for transplant in a timely manner. While the three-part ISHLT consensus documents on lung transplantation in patients with systemic autoimmune rheumatic diseases provide an excellent framework [14, 16, 17], we still lack a structured, phenotype-specific perioperative strategy for conditions such as anti-MDA5 RP-ILD. This underscores a critical unmet need in lung transplantation candidate selection and management in IIM population. To objectively evaluate and optimize long-term outcomes, future prospective studies must develop transplant guidelines and employ standardized protocols for both pre-transplant assessment, including immunosuppressive management and ECMO bridging, and post-transplant monitoring for complications such as myositis recurrence, infection and rejection.

## Supplementary Material

keag200_Supplementary_Data

## Data Availability

The data that support the findings of this study are available from the corresponding author upon reasonable request.
