# Supplementary material for: Lung transplant outcomes in myositis, systemic sclerosis and idiopathic pulmonary fibrosis: a multicentre retrospective analysis
Source: Rheumatology (Oxford). 2026 Apr 15;65(5):keag200. doi: 10.1093/rheumatology/keag200 (PMC13180644; doi:10.1093/rheumatology/keag200)

**Supplementary** **Table S1: Serological profile of patients with IIM and SSc**

| Serology | IIM (n=22) | SSc (n=32) |
| --- | --- | --- |
| Anti-MDA5 | 10 | 0 |
| Anti-Ro52 | 12 | 1 |
| Anti-Jo-1 | 6 | 0 |
| Anti-PL12 | 1 | 0 |
| Anti-OJ | 1 | 0 |
| Anti-Ku | 1 | 0 |
| Anti-PM/Scl | 2 | 0 |
| Anti-TIF 1 gamma | 1 | 0 |
| Anti-Mi2 | 4 | 0 |
| Anti-SAE | 1 | 0 |
| Anti-SRP | 1 | 0 |
| Anti-HMGCR | 1 | 0 |
| Anti-MUP44 | 1 | 0 |
| Anti-SSA | 5 | 6 |
| Anti-SSB | 0 | 1 |
| Anti-Scl-70 | 0 | 12 |
| Anti-CENP-B | 0 | 2 |
| Anti-RNA pol-III | 0 | 1 |
| Anti-U1-RNP | 1 | 4 |
| Anti-Sm | 0 | 3 |
| Anti-dsDNA | 0 | 1 |

**Supplementary** **Table S2: Other clinical variables pre-transplant**

| Variables before Transplant | IIM (n=22) | SSc (n=32) | IPF (n=64) |
| --- | --- | --- | --- |
| Duration of mechanical ventilation, days, median [IQR] | 23  [10 – 36] | 6  [6– 7] | 15  [15 – 29] |
| Duration of ECMO, days, Median [IQR] | 19 [16 – 34] | 31 [17– 45] * | 9* |
| New renal replacement therapy, n (%) | 3 (13.6) | 0 | 0 |
| CMV mismatch Donor (+) / Recipient (-), n (%) | 4 (18) | 3 (9) | 9 (14) |
| Sepsis, n (%) | 3 (14) | 0 | 1 (2) |

*ECMO duration is based on 2 SSc patients and 1 IPF patient.

CMV: Cytomegalovirus. ECMO: Extracorporeal Membrane Oxygenation

**Supplementary** **Table S3: Immunosuppression regimens for post-transplant maintenance**

| Post-transplant Anti-Rejection | IIM (n=22) | SSc (n=32) |
| --- | --- | --- |
| Tacrolimus (%) | 22 (100) | 32 (100) |
| Mycophenolate mofetil (%) | 21 (95) | 31 (97) |
| Prednisone (%) | 22 (100) | 32 (100) |
| Azathioprine (%) | 0 (0) | 2 (6) |
| Sirolimus (%) | 2 (9) | 0 (0) |

**Supplementary** **Figure S1: Summary of clinical manifestations in IIM (A) and SSc (B) patients at presentation.**

A.

**B**

CK: Creatine Kinase; ULN: Upper Limit of Normal; DM: Dermatomyositis; EMG: Electromyography; GERD: Gastroesophageal Reflux Disease; SIBO: Small Intestinal Bacterial Overgrowth.

**Supplemental Figure S2: Kaplan-Meier Survival curve of IIM patients stratified by (A) emergency vs. non-emergency transplant, (B) pre-transplant ECMO status, and (C) amyopathic vs. myopathic IIM subtypes.**

**
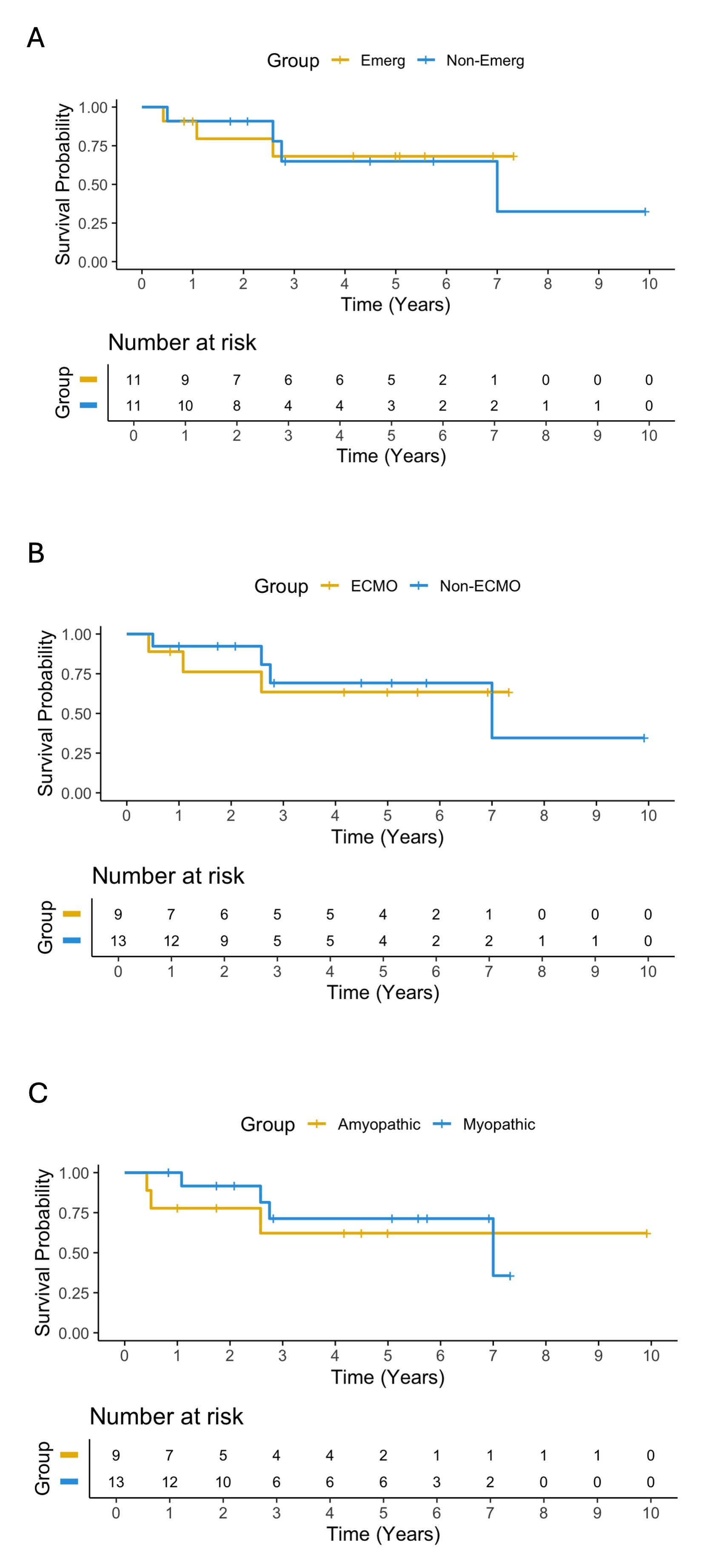
**

**Supplemental Figure S3: Kaplan-Meier Survival curve of SSc patients stratified by (A) presence of severe PH, (B) emergency vs non-Emergency transplant, and (C) presence of esophageal dysmotility.**


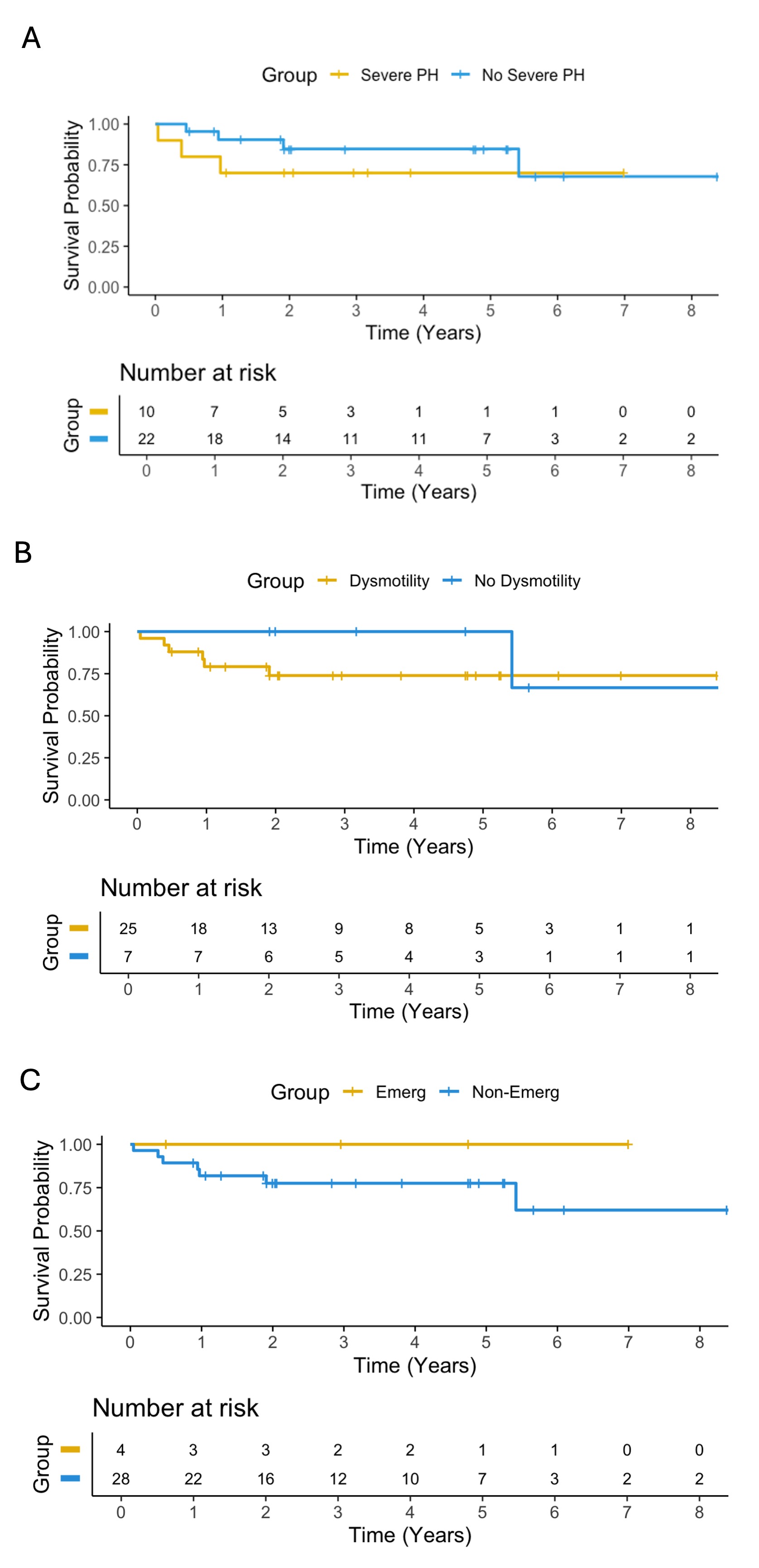

Supplement: keag200_Supplementary_Data [file keag200_supplementary_data.docx]
